# Prophylactic cranial irradiation (PCI) versus active surveillance in patients with limited-stage small cell lung cancer: a retrospective, multicentre study

**DOI:** 10.1186/s12931-022-02196-2

**Published:** 2022-10-02

**Authors:** Yu Chen, Yao Wang, Fei Ren, Zhaoqin Huang, Bingxu Tan, Zhonghua Zhao, Xinshuang Yu, Peng Dong, Jinming Yu, Xiangjiao Meng

**Affiliations:** 1 Shandong University Cancer Center, Jinan, Shandong China; 2 Department of Radiation Oncology, Shandong Cancer Hospital and Institute, Shandong First Medical University and Shandong Academy of Medical Sciences, Jiyan Road 440, Jinan, 250117 Shandong China; 3 Department of Radiology, Shandong Provincial Hospital Affiliated to Shandong First Medical University, Jinan, Shandong China; 4 Department of Radiation Oncology, Qilu Hospital of Shandong University, Jinan, Shandong China; 5 Department of Oncology, Binzhou Medical University Hospital, Binzhou, Shandong China; 6 Shandong Key Laboratory of Rheumatic Disease and Translational Medicine, Department of Oncology, The First Affiliated Hospital of Shandong First Medical University & Shandong Provincial Qianfoshan Hospital, Shandong Lung Cancer Institute, Jinan, Shandong China; 7 Shandong Provincial Key Laboratory of Radiation Oncology, Department of Radiation Oncology, Shandong Cancer Hospital and Institute, Shandong First Medical University and Shandong Academy of Medical Sciences, Jinan, Shandong China; 8 8Research Unit of Radiation Oncology, Chinese Academy of Medical Sciences, Jinan, Shandong China

**Keywords:** Prophylactic cranial irradiation, Surveillance, Limited-stage small cell lung cancer, MRI, Survival

## Abstract

**Background:**

The recommendation of PCI for limited-stage small cell lung cancer (LS-SCLC) is primarily based on evidence from the pre-magnetic resonance imaging (MRI) era. However, as MRI accuracy improves and stereotactic radiosurgery advances, the role of PCI for LS-SCLC has become uncertain. This study aims to compare the contemporary survival outcomes of patients with LS-SCLC treated with PCI versus active surveillance.

**Methods:**

We conducted a retrospective cohort study in which 1068 patients with LS-SCLC who achieved a good response to first-line chemoradiotherapy were consecutively enrolled from 5 tertiary medical centres between June 2009 and June 2019. Of these patients, 440 received PCI, while 628 received surveillance without PCI. Propensity score matching with a 1:1 ratio was performed to balance the baseline characteristics of the two cohorts. The endpoints were overall survival (OS) and the incidence of brain metastasis (BM).

**Results:**

In total, 648 patients were matched. The baseline characteristics were generally well balanced. At a median follow-up of 64.5 months (range 2–190), patients who underwent PCI had a significantly lower risk for BM than those who underwent surveillance. The 3-year cumulative incidence rate of BM was 28.2% (95% CI 22.5–33.8%) in the PCI cohort and 38.5% (32.6–44.5%) in the surveillance cohort (Gray’s *p* = 0.002). However, the lower incidence of BM in the PCI cohort did not translate into a significant extension of OS. The median OS was 35.8 months (95% CI 27.6–44.0 months) in the PCI cohort versus 32 months (26.4–37.6 months) in the surveillance cohort (HR 0.90, 95% CI 0.74–1.10, *p* = 0.29). Multivariable analysis showed that disease stage, chemoradiotherapy sequence, and response to chemoradiotherapy were independent prognostic factors for BM or OS.

**Conclusions:**

Overall, PCI reduces the risk for BM but does not substantially prolong OS compared with active surveillance. A phase 3, prospective clinical trial (NCT04829708) we initiated is currently underway, which is expected to corroborate our results.

## Background

Prophylactic cranial irradiation (PCI) remains the standard recommendation for LS-SCLC with a good response to first-line chemoradiotherapy (CRT), but this suggestion is primarily based on a large meta-analysis conducted before the MRI era that showed a 5.4% overall survival (OS) benefit with PCI for LS-SCLC [1, 2]. Generally, SCLC, characterized by rapid growth and early dissemination, has a higher propensity for brain metastasis than many other solid tumours, with an incidence of BM within 2 years of up to 50–60% [3, 4]. Historically, BMs are usually accompanied by devastating complications, resulting in an appreciably decreased quality of life (QoL) and shortened OS [5, 6]. PCI, with the aim of eradicating all potential subclinical lesions in the brain, is preferred in LS-SCLC management based on several previous studies that consistently demonstrated decreased BM rates and improved survival benefits with PCI [2, 7]. However, as medical technology and discoveries evolve and advance, this dogma is being challenged. A multicentre randomized trial of extensive-stage SCLC (ES-SCLC) in Japan was the first to demonstrate that PCI omission did not compromise survival benefits and even had a trend towards improved OS (median OS PCI vs. PCI omission, 13.7 vs. 11.6 months, p = 0.09) in the contemporary era with periodic MRI examinations [8].

The evolving role of PCI in ES-SCLC and contemporary advances in technology and treatment modalities have made preference for PCI in LS-SCLC to become uncertain. There are several issues to be considered. First, it is speculated that the previously proven benefits of PCI from the pre-MRI era may be magnified by mixing a subset of patients already with asymptomatic BM [9]. However, contemporary MRI, which has remarkably improved fidelity, can eliminate this potential bias [10] and challenges the preference for PCI. Second, stereotactic radiosurgery (SRS) has become a well-established front-line therapy for limited BM. To date, although SRS for BM is still not a standard of treatment and is mainly used in some selected patients, several studies have demonstrated comparable OS and substantially decreased neuropsychological sequelae compared with whole brain radiotherapy (WBRT) [11–13]. Active MRI surveillance facilitates the early detection of patients with limited BM, further allowing for effective salvage SRS, and free from compulsory radiation to the entire brain, which has important implications when reconsidering the role of PCI. Third, the recommended first-line CRT regimen has been gradually standardized with relatively improved survival benefits [14]. Exploration of the role of PCI in contemporary standard regimens is warranted. Furthermore, novel treatment modalities, including immunotherapy, have been explored in multiple ongoing clinical trials and are expected to improve prognosis and possibly reduce the incidence of BM [15–17]. Thus, the role of PCI is worth exploring.

Several retrospective studies with small sample sizes have explored the role of PCI for LS-SCLC in the MRI era with inconsistent results [18–22]. We speculated that PCI can indeed offer a consistent reduction in the incidence of BM but fail to translate into significantly improved OS when compared with active surveillance. In the present study, we retrospectively analysed a large sample-sized cohort from multiple tertiary hospitals to compare the OS and incidence of BM between patients treated with PCI and active surveillance.

## Methods

### Study design and patients

This retrospective cohort study enrolled consecutive patients with LS-SCLC from 5 tertiary medical centres between June 2009 and June 2019. Patients were included for analysis if they achieved a complete response (CR) or partial response (PR) after first-line CRT. Brain MRI at baseline and before PCI was required to exclude patients who had already developed BM. Brain CT with contrast was allowed if MRI was contraindicated or inconvenient. Patients were excluded if they received fewer than four cycles of chemotherapy, developed BM within 1 month after CRT, or were lost to follow-up after CRT and PCI. Detailed data on the baseline characteristics, therapeutic regimens, radiographic findings, survival results and others were extracted from electronic medical records. Patients underwent hospital follow-up with clinical and radiographic surveillance, as determined per institution. The standard follow-up schedule was every 3 months in the first 2 years and then every 6 months until brain metastases occurred or the patient died. The radiographic response assessment was performed in accordance with the Response Evaluation Criteria in Solid Tumors (RECIST), version 1.1 [23]. The study was conducted in accordance with the Declaration of Helsinki and approved by the independent ethics committees or institutional review boards of each participating medical centre.

### Treatment

Patients were administered 4 to 6 cycles of etoposide-platinum chemotherapy in 3-week cycles with specific medication according to the Chinese Society of Clinical Oncology (CSCO) guidelines or National Comprehensive Cancer Network (NCCN) guidelines. Definitive thoracic radiotherapy (TRT) was delivered concurrently or sequentially with chemotherapy, mainly depending on the size of the tumour and patient tolerance. TRT was delivered with intensity-modulated radiotherapy or three-dimensional conformal radiotherapy techniques. The majority of patients were treated with standard dose/fractionation regimens of either 45 Gy in 30 fractions (hyper-fractionated, twice daily) or 60 to 66 Gy in 30 to 33 fractions (conventional fractionated, once daily). PCI was typically conducted within 6 weeks after completion of first-line CRT if tumour restaging examinations at that time indicated a good response from intrathoracic disease and no evidence of distant metastasis. PCI at a dose of either 25 Gy in 10 fractions (2.5 Gy per fraction, 5 days per week) or 30 Gy in 10 fractions (3 Gy per fraction, 5 days per week) was recommended.

### Outcomes

The endpoints of this analysis included OS and incidence of BM. OS was defined as the time from the end of first-line CRT to the date of death from any cause or last follow-up visit. The cumulative incidence of BM was defined as the proportion of patients who developed BM during follow-up, with death as a competing event. The cumulative incidence of BM at 1 year, 3 years and 5 years was calculated. Exploratory subgroup analysis was performed based on several covariates to explore the potential population who would benefit from such treatment.

### Statistical analysis

Descriptive statistics were used to show the distribution of baseline and therapeutic characteristics of the patients, with counts (percentages) for categorical variables and medians (interquartile range) for continuous variables. Propensity score matching was performed to adjust unbalanced covariates between the two cohorts. A multivariable logistic regression model was used to calculate the propensity score for each enrolled patient based on several clinically relevant covariates, including age, gender, Eastern Cooperative Oncology Group performance status (ECOG PS), smoking status, tumour stage at diagnosis, surgery history, CRT sequence, and response to CRT (CR or PR). Patients in the two cohorts were then matched by nearest neighbour matching in a 1:1 ratio. A calliper of 0.2 was applied as the maximum tolerated difference of the paired propensity score. Baseline characteristics between the two cohorts were compared using chi-square tests for categorical variables and t tests for continuous variables.

OS was estimated by Kaplan–Meier curves and compared by log-rank tests. Competing risk regression analysis was used to calculate the cumulative incidence of BM in which death without BM was counted as a competing risk. The cumulative incidence curves showed the cumulative risk of BM over time, while Gray’s tests were used to compare the difference between the two cohorts. The exploratory subgroup analyses were performed with unstratified Cox proportional hazards models with estimated hazard ratios (HRs) and associated 95% confidence intervals (CIs). The results are presented in a forest plot. In addition, univariate and multivariable Cox analyses and Fine-Gray model analyses were performed to identify significant prognostic factors for OS and BM.

A two-sided *p* value less than 0.05 was considered statistically significant. All statistical analyses were performed using R version 4.1.2 (R Project for Statistical Computing) and SPSS version 23.0 (IBM Corporation, Armonk, NY, USA).

## Results

### Patient characteristics

**Fig. 1:**
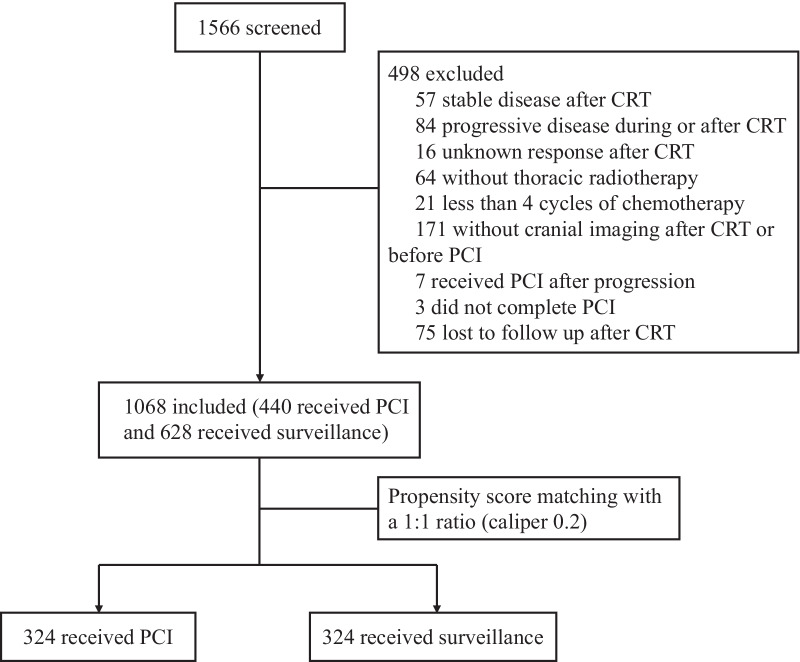
Flowchart of patient enrollment. *CRT* chemoradiotherapy, *PCI* prophylactic cranial irradiation

Between June 2009 and June 2019, 1068 patients with LS-SCLC who achieved a good response to first-line CRT were consecutively enrolled in this study; among these patients, 440 received PCI (Fig. 1). The baseline and therapeutic characteristics are summarized in Table 1*.* Overall, the majority were male (69.9%) and had a good performance status (ECOG 0–1 96.0%). Most patients (82.2%) had stage III disease at the time of diagnosis, and 96.3% of patients received etoposide + platinum chemotherapy. Patients in the PCI cohort were relatively younger than those in the surveillance cohort, with a median age of 57 (IQR 51–63) vs. 59 (52–64). The proportion of patients with concurrent CRT was significantly higher in the PCI cohort than in the surveillance cohort (44.8% vs. 28.0%, *p* < 0.001). In addition, 32.7% of patients in the PCI cohort achieved CR after CRT, while 26.1% achieved CR in the surveillance cohort (*p* = 0.019).

**Table 1:** Baseline and therapeutic characteristics of patients before and after propensity score matching

Characteristics	Before matching (%)			After matching (%)		
	PCI (n = 440)	Active surveillance (n = 628)	*p* value	PCI (n = 324)	Active surveillance (n = 324)	*p* value
Age, median (IQR), y	57 (51–63)	59 (52–64)	0.029	58 (51–64)	59 (51–65)	0.268
< 65	356 (80.9)	473 (75.3)	0.031	255 (78.7)	240 (74.1)	0.165
≥ 65	84 (19.1)	155 (24.7)		69 (21.3)	84 (25.9)	
Gender						
Male	312 (70.9)	434 (69.1)	0.528	232 (71.6)	229 (70.7)	0.795
Female	128 (29.1)	194 (30.9)		92 (28.4)	95 (29.3)	
ECOG PS						
0	52 (11.8)	91 (14.5)	0.427	41 (12.7)	39 (12)	0.609
1	371 (84.3)	511 (81.4)		272 (84)	269 (83)	
2	17 (3.9)	26 (4.1)		11 (3.4)	16 (4.9)	
Smoking status						
Never	188 (42.7)	263 (41.9)	0.831	138 (42.6)	139 (42.9)	0.762
Former	40 (9.1)	64 (10.2)		27 (8.3)	32 (9.9)	
Current	212 (48.2)	301 (47.9)		159 (49.1)	153 (47.2)	
Disease stage						
I–II	88 (20)	102 (16.2)	0.114	62 (19.1)	54 (16.7)	0.412
III	352 (80)	526 (83.8)		262 (80.9)	270 (83.3)	
Surgery						
Yes	31 (7)	54 (8.6)	0.356	29 (9)	28 (8.6)	0.89
No	409 (93)	574 (91.4)		295 (91)	296 (91.4)	
Chemotherapy						
Etoposide + platinum	428 (97.3)	601 (95.7)	0.12	315 (97.2)	309 (95.4)	0.212
Others	11 (2.5)	27 (4.3)		9 (2.8)	15 (4.6)	
CRT sequence						
Sequential	243 (55.2)	452 (72)	< 0.001	209 (64.5)	192 (59.3)	0.169
Concurrent	197 (44.8)	176 (28)		115 (35.5)	132 (40.7)	
Response to CRT						
PR	296 (67.3)	464 (73.9)	0.019	233 (71.9)	225 (69.4)	0.49
CR	144 (32.7)	164 (26.1)		91 (28.1)	99 (30.6)	

*PCI* prophylactic cranial irradiation, *IQR* interquartile range, *y* years, *ECOG PS* Eastern Cooperative Oncology Group performance status, *CRT* chemoradiotherapy, *CR* complete response, *PR* partial response

After propensity score matching, 324 patients were eventually matched in each cohort. The baseline and therapeutic characteristics were generally well balanced between the two matched cohorts (Table 1).

### Survival outcomes

**Fig. 2:**
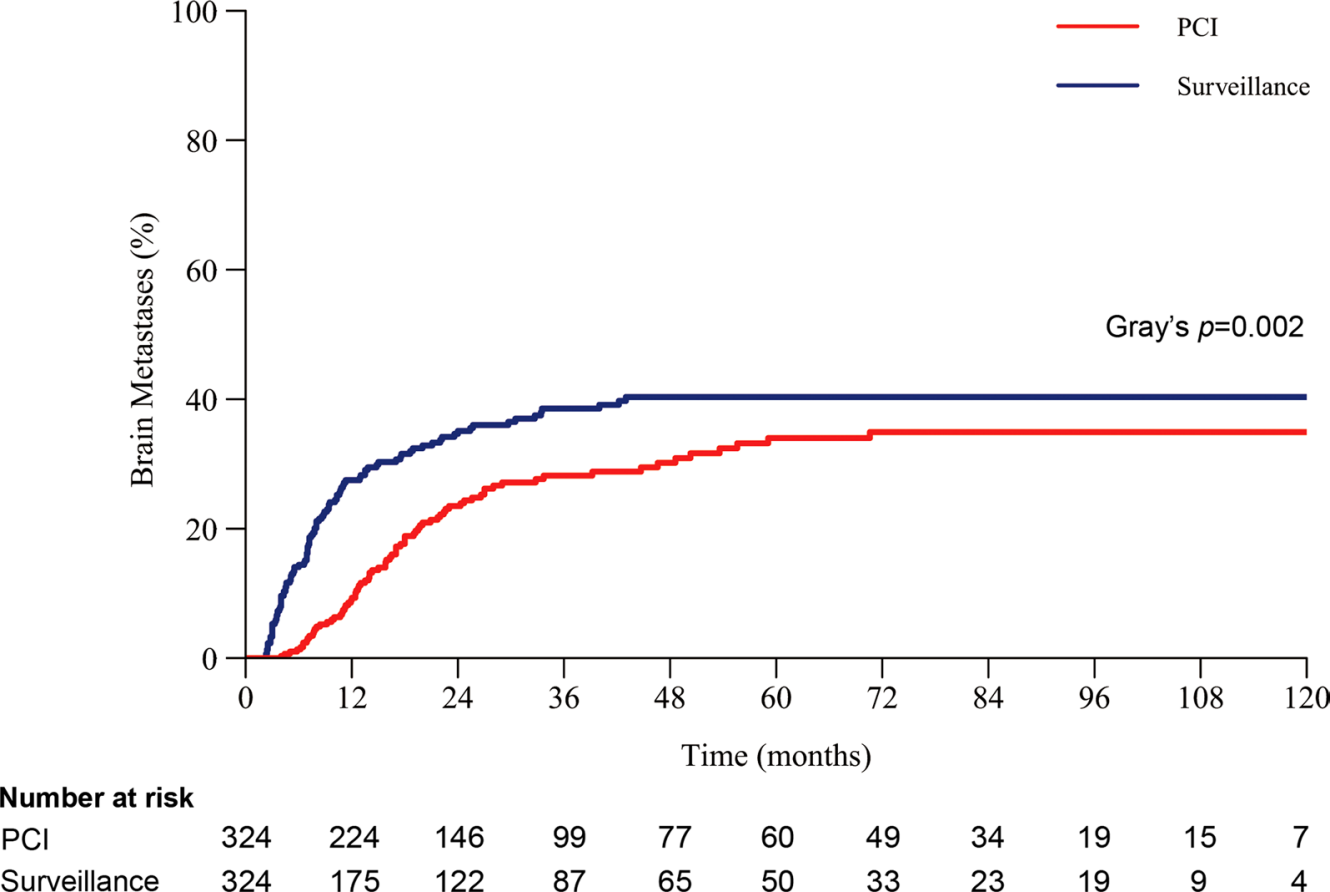
Cumulative incidence of BM (with death as competing risk). *PCI* prophylactic cranial irradiation

At a median follow-up of 64.5 months (61.6 months in the surveillance cohort vs. 70.9 months in the PCI cohort, *p* = 0.764), 186 patients developed BM, of which 106 patients were included in the surveillance cohort. The cumulative incidence of BM was significantly higher in the surveillance cohort than in the PCI cohort when death was counted as a competing risk (Gray’s *p* = 0.002, Fig. 2). The 1-year, 3-year, and 5-year cumulative incidence rates of BM in the surveillance and PCI cohorts were 27.5% (95% CI 22.3–32.7%) vs. 9.3% (5.9–12.7%), 38.5% (32.6–44.5%) vs. 28.2% (22.5–33.8%), and 40.3% (34.2–46.5%) vs. 34.0% (27.5–40.5%), respectively. Fifty-two of the 106 patients (49.1%) who developed BM in the surveillance cohort had no relative symptoms when imaging confirmed the presence of BM, while 31 of the 80 patients (38.8%) in the PCI cohort were diagnosed with asymptomatic BM. Among the patients with BM, 91.5% of patients in the surveillance cohort received salvage brain radiotherapy, compared with only 58.8% in the PCI cohort. The modalities of salvage brain radiotherapy varied, including WBRT only, WBRT plus sequential or simultaneous integrated boost, conventional radiotherapy for the brain metastases only, and SRS. Only a small number of patients received SRS, including 7.5% of patients in the surveillance cohort and 8.8% in the PCI cohort.

**Fig. 3:**
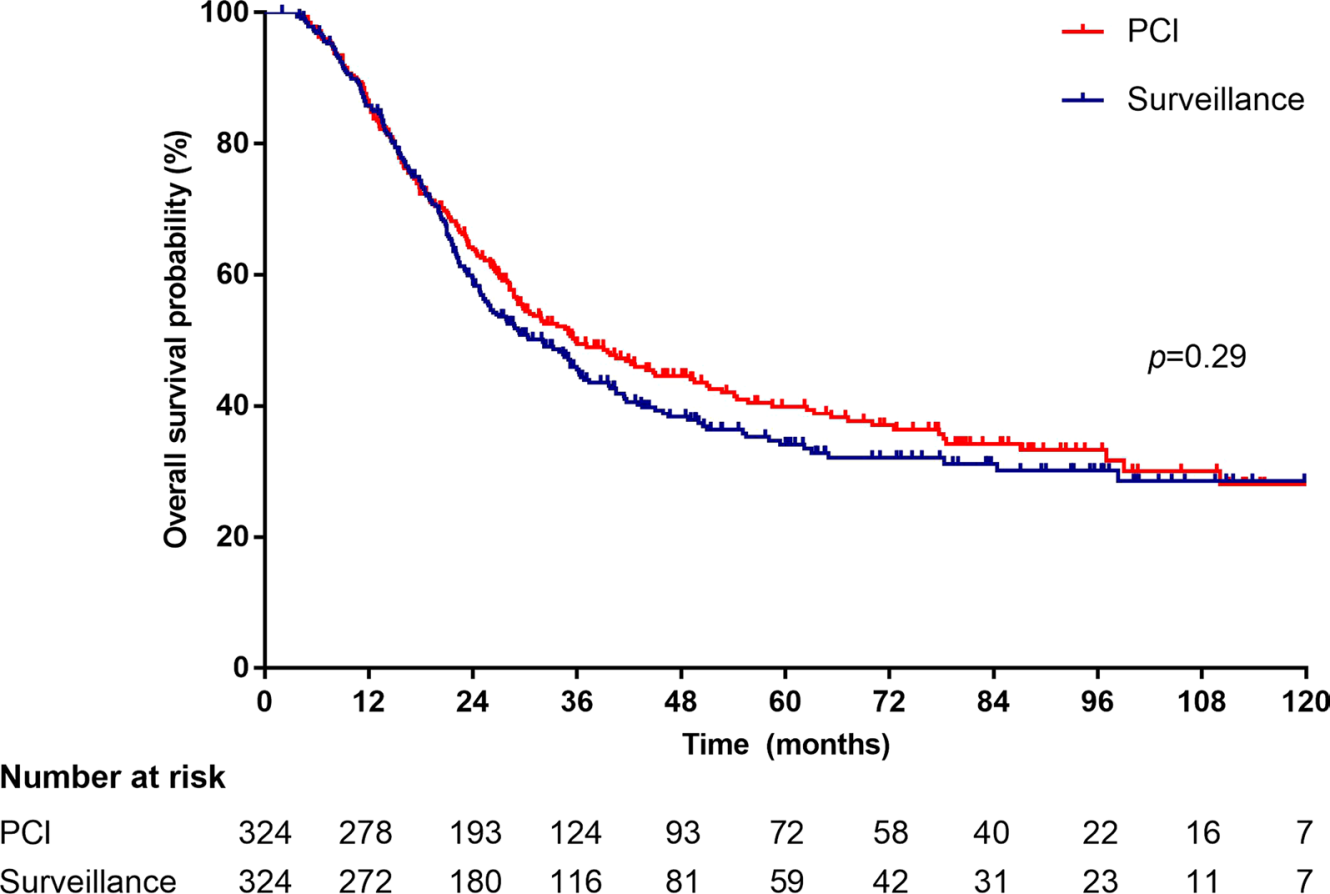
Kaplan–Meier analysis of OS. *PCI* prophylactic cranial irradiation

**Fig. 4:**
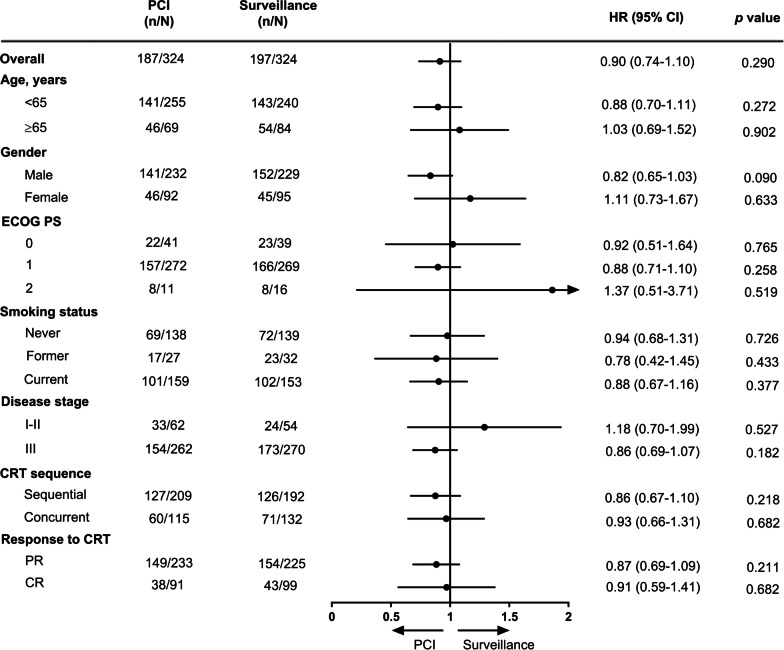
Exploratory subgroup analysis of OS. *PCI* prophylactic cranial irradiation, *HR* hazard ratio, *ECOG PS* Eastern Cooperative Oncology Group performance status, *CRT* chemoradiotherapy, *CR* complete response, *PR* partial response

The lower incidence of BM in the PCI cohort did not translate to a substantial extension of OS. The median OS was 35.8 months (95% CI 27.6–44.0 months) in the PCI cohort versus 32 months (95% CI 26.4–37.6 months) in the surveillance cohort (HR 0.90, 95% CI 0.74–1.10, *p* = 0.29, Fig. 3). The 1-year, 3-year, and 5-year OS rates in the PCI and surveillance cohorts were 86.6% (82.9–90.3%) vs. 85.8% (81.9–89.7%), 49.4% (43.7–55.1%) vs. 45.5% (39.8–51.2%), 39.9% (34.0–45.8%) vs. 34.1% (28.2–40.0%), respectively. Further exploratory subgroup analyses of OS did not find any factors favouring PCI (Fig. 4). OS was not significantly different between the two cohorts among several key subgroups.

The median progression-free survival (PFS) was substantially longer with PCI (14.7 months, 95% CI 11.5–17.9 months) than with surveillance (10.0 months, 95% CI 7.8–12.2 months), with an HR of 0.74 (95% CI 0.61–0.90, p = 0.002). A total of 410 patients developed disease progression during follow-up, of which 192 patients were included in the PCI cohort. At the time of confirmed disease progression, 84 patients had only BM (22 of 192 [11.5%] in the PCI cohort vs. 62 of 218 [28.4%] in the surveillance cohort); 23 patients had BM combined with extracranial progression (6 of 192 [3.1%] vs. 17 of 218 [7.8%]); and 303 patients had only extracranial progression (164 of 192 [85.4%] vs. 139 of 218 [63.8%]).

### Prognostic analysis of OS and BM

Multivariable analysis of prognostic factors for OS showed that concurrent CRT and CR after CRT were significantly associated with improved OS (HR 0.66, 95% CI 0.53–0.83, *p* = 0.005; HR 0.42, 95% CI 0.32–0.54, *p* < 0.001, respectively; Table 2). In addition, multivariable analysis of prognostic factors for BM indicated that PCI, stage I–II disease, and CR after CRT were independently associated with significantly lower rates of BM (HR 0.60, 95% CI 0.45–0.80, *p* < 0.001; HR 0.63, 95% CI 0.40–0.99, *p* = 0.047; HR 0.58, 95% CI 0.40–0.84, *p* = 0.004, respectively; Table 2). Other factors, including age, gender, ECOG PS, and smoking status, were not found to have independent correlations with OS and BM.

**Table 2:** Univariable and multivariable analyses of BM and OS

Characteristics	Univariable (BM)	Multivariable (BM)		Univariable (OS)	Multivariable (OS)	
	*p* value	HR (95% CI)	*p* value	*p* value	HR (95% CI)	*p* value
Age, y	0.881			0.013		
< 65		1 (ref)			1 (ref)	
≥ 65		1.01 (0.70–1.47)	0.95		1.13 (0.88–1.45)	0.349
Gender	0.099			0.001		
Male		1 (ref)			1 (ref)	.
Female		0.79 (0.51–1.23)	0.29		0.80 (0.59–1.09)	0.156
ECOG PS	0.875			0.119		
0		1 (ref)			1 (ref)	
1		1.27 (0.82–1.95)	0.28		1.02 (0.74–1.42)	0.897
2		1.67 (0.66–4.18)	0.28		1.07 (0.58–1.96)	0.832
Smoking status	0.277			0.001		
Never		1 (ref)			1 (ref)	
Former		1.07 (0.59–1.95)	0.82		1.34 (0.90–2.00)	0.148
Current		0.91 (0.61–1.38)	0.66		1.26 (0.95–1.66)	0.108
Disease stage	0.004			0.017		
I-II		1 (ref)			1 (ref)	
III		1.59 (1.01–2.50)	0.047		1.04 (0.77–1.40)	0.798
CRT sequence	0.351			0.005		
Sequential		1 (ref)			1 (ref)	
Concurrent		0.79 (0.58–1.07)	0.13		0.66 (0.53–0.83)	< 0.001
Response to CRT	0.001			< 0.001		
PR		1 (ref)			1 (ref)	
CR		0.58 (0.40–0.84)	0.004		0.42 (0.32–0.54)	< 0.001
PCI	0.002			0.291		
No		1 (ref)			1 (ref)	
Yes		0.60 (0.45–0.80)	< 0.001		0.85 (0.69–1.04)	0.112

*BM* brain metastases, *OS* overall survival, *HR* hazard ratio, *CI* confidence interval, *y* years, *ECOG PS* Eastern Cooperative Oncology Group performance status, *CRT* chemoradiotherapy, *CR* complete response, *PR* partial response, *PCI* prophylactic cranial irradiation

## Discussion

This is the largest multicentre, retrospective cohort study, to our knowledge, to explore the role of PCI for patients with LS-SCLC who achieved a good response to first-line CRT in the contemporary MRI era. The findings demonstrated that PCI did appreciably reduce the risk of BM. However, the lower incidence of BM did not translate to a significant gain in OS. There was no statistically significant difference in OS between the PCI cohort and surveillance cohort in the matched population or among several key subgroups. PCI was associated with improved PFS, which might be attributed to the reduction in BM but not extracranial metastasis. Multivariable analysis indicated that disease stage, CRT sequence, and response to CRT were independent prognostic factors for BM and OS, providing an important reference and basis for establishing stratification factors in future clinical trials.

MRI, which has high fidelity, has led to a paradigm shift in brain management and is now recommended as a preferred means to detect BM in patients with SCLC [10, 24]. The conflicting survival outcomes in the EORTC trial and Japanese trial of ES-SCLC challenged the contemporary rationality to recommend PCI for LS-SCLC, which was mainly based on pre-MRI data [7, 8]. Several small retrospective studies have explored the role of PCI for LS-SCLC in the contemporary era [18–22]. Nevertheless, the findings varied. Michael Yan et al. reported the outcomes of PCI for LS-SCLC over a 20-year period at the Princess Margaret Cancer Centre. The findings showed that PCI contributed to improved OS and lower BM risk (HR 1.88, 95% CI 1.32–2.69; HR 4.66, 95% CI 2.58–8.40, respectively) [21]. Conversely, in a recent analysis of 297 patients with LS-SCLC, PCI was not independently associated with substantial improvement in OS (HR 0.844, 95% CI 0.604–1.180, *p* = 0.32) [18]. S. Ghanta et al. demonstrated that PCI significantly prolonged neurological survival (HR 0.23, 95% CI 0.08–0.65; *p* = 0.01) and brain metastasis-free survival (HR 0.25, 95% CI 0.12–0.51; *p* < 0.01) but had no role in improving OS (HR 0.74, 95% CI 0.49–1.11; *p* < 0.01) [19]. We included 1068 patients with LS-SCLC, including 324 in each cohort who were matched for further comparisons. The findings revealed that PCI led to a consistent reduction in BM rate, but this reduction failed to translate into significantly improved OS. This analysis, which has the largest sample size thus far, offers an important reference for future exploration.

It was previously thought that BM often leads to devastating complications, further reduced QoL and shortened OS [5, 6]. However, the significant decrease in BM rate in this analysis did not result in an appreciably improved OS, which might be attributed to the early and effective salvage therapy adopted to treat BM. The application of MRI promotes early detection of asymptomatic BM when the lesions are usually localized and have a low tumour burden [10, 25]. Further timely and effective salvage irradiation could eradicate BM lesions without affecting the control of systemic diseases. In this analysis, 49.1% of patients who developed BM in the surveillance cohort were asymptomatic at the time of imaging confirmation, while 38.8% were asymptomatic in the PCI cohort. Salvage cranial irradiation was conducted in 91.5% of patients in the surveillance cohort compared with only 58.8% in the PCI cohort. We speculate that one of the main reasons for the lower proportion of patients receiving salvage irradiation after BM in the PCI cohort was that some patients were unfit to receive cranial irradiation again, and because of the poor effects of systemic treatment, these patients could not receive effective therapy, thus leading to poor prognosis.

Typically, it is the risk–benefit trade-off that helps to determine the feasibility of a particular treatment. If the reduction in BM contributes to improved QoL and stable cognition, PCI could be reasonably recommended for patients with LS-SCLC, even without significant OS benefits. However, several previous studies have revealed that PCI was associated with appreciable neurocognitive toxicity and worsened QoL [26–28]. A pooled secondary analysis of the Radiation Therapy Oncology Group (RTOG) 0212 and 0214 trials demonstrated that the risk of decline in patient-reported cognitive functioning was elevated at least threefold at both 6 and 12 months following PCI compared to after surveillance [26]. Le Pechoux et al. reported mild neurocognitive deterioration over time in patients who underwent PCI, including communication deficits, weakness of the legs, intellectual deficits and memory loss [27]. Consequently, approximately 40% of patients with LS-SCLC refused to receive PCI for fear of neurocognitive toxicity [9, 29, 30]. Hippocampal-avoidance PCI has been explored, expecting to preserve cognitive function without sacrificing OS and intracranial control. However, two prospective trials showed inconsistent outcomes regarding whether a reduced influence on neurocognitive function could be achieved with hippocampal-avoidance PCI compared to conventional PCI [31, 32]. The present study was a retrospective analysis of the survival results in PCI and surveillance cohorts and lacked complete information to evaluate the neurocognitive toxicity of PCI and hippocampal-avoidance PCI. The noninferiority PRIMALung Study conducted by EORTC (NCT04790253) and a phase 3, prospective clinical trial (NCT04829708) we initiated are currently underway, which might provide definite evidence for future reference.

Advances in medical technology and the development of novel treatment regimens have also challenged the recommendation of PCI for LS-SCLC. SRS, as a well-established treatment modality, has been applied as first-line therapy for limited BM in various solid tumours and achieved comparable OS and decreased rates of neurocognitive sequelae compared with WBRT [11–13]. However, concerns exist regarding the use of SRS alone for SCLC because of the possibility of subsequent diffuse central nervous system (CNS) progression, increased neurologic mortality, and need for more BM salvage therapy [9, 33]. Promisingly, a growing number of retrospective studies have indicated that SRS has superior benefits over WBRT for recurrent BM in SCLC after PCI and BM without prior PCI or WBRT [13, 34–36]. Nonetheless, the role of first-line SRS in contemporary SCLC management remains unclear without definite evidence. In the present study, less than 10% of patients with BM in the surveillance cohort received SRS as salvage therapy. Prospective clinical trials are warranted to confirm the potential practice-changing advances. The success of immunotherapy in ES-SCLC has promoted its exploration in LS-SCLC [15, 37]. Welsh et al. conducted a phase I/II trial of pembrolizumab with concurrent CRT for LS-SCLC. The results demonstrated favourable outcomes with well-tolerated toxicity [16]. In addition, improved posterior-line therapy for LS-SCLC led to longer survival than before. Re-exploration of the role of PCI for LS-SCLC is desperately needed in the context of increasing available modern advances.

Clarifying the population that could benefit from PCI has considerable significance when weighing optimal treatment options. There are perspectives suggesting that patients with a high risk of BM would benefit from PCI. Multivariable analysis in the present study showed that stage III disease and PR after CRT were independent risk factors for BM. Unexpectedly, the subgroup analysis did not find that PCI was significantly superior to surveillance in terms of OS. We speculate that active MRI surveillance and early effective salvage therapy might not be inferior to PCI, even in patients at high risk for BM. Large randomized clinical trials are warranted to test this hypothesis.

The study has several limitations. First, this is a retrospective cohort analysis with potential selection bias. To minimize the impact of possible confounding factors, we consecutively enrolled patients from 5 tertiary medical centres. Propensity score matching was performed to balance the baseline characteristics between the two cohorts. Nevertheless, the results should be interpreted with caution. Second, cognitive function and QoL were unavailable for analysis due to the retrospective nature of the study. Furthermore, the surveillance practices were mainly determined at the discretion of the clinicians and were dependent on patient compliance. Moreover, the surveillance follow-ups of some patients were irregular. A number of patients were lost to follow-up after several visits, which might have affected the assessment of the cumulative incidence of BM.

## Conclusions

In summary, this cohort analysis indicated that PCI led to a consistent reduction in BM rate but did not substantially prolong OS compared with active surveillance. The results challenge the standard recommendation of PCI for LS-SCLC in contemporary practice. Disease stage, CRT sequence, and response to CRT were demonstrated to be independent prognostic factors for BM or OS, with important implications for establishing stratified factors in future clinical trials. A phase 3, prospective clinical trial (NCT04829708) we initiated is currently underway, which is expected to validate our results.

## Data Availability

The datasets used and/or analysed during the current study are available from the corresponding author on reasonable request.

## Abbreviations

PCI: Prophylactic cranial irradiation
LS-SCLC: Limited-stage small cell lung cancer
MRI: Magnetic resonance imaging
OS: Overall survival
BM: Brain metastases
CI: Confidence interval
HR: Hazard ratio
CRT: Chemoradiotherapy
QoL: Quality of life
ES-SCLC: Extensive-stage small cell lung cancer
SRS: Stereotactic radiosurgery
WBRT: Whole brain radiotherapy
CR: Complete response
PR: Partial response
CT: Computed tomography
RECIST: Response Evaluation Criteria in Solid Tumors
CSCO: Chinese Society of Clinical Oncology
NCCN: National Comprehensive Cancer Network
TRT: Thoracic radiotherapy
IQR: Interquartile range
ECOG PS: Eastern Cooperative Oncology Group performance status
PFS: Progression-free survival
RTOG: Radiation Therapy Oncology Group
CNS: Central nervous system
